# Frequency of the *C9orf72* hexanucleotide repeat expansion in patients with amyotrophic lateral sclerosis and frontotemporal dementia: a cross-sectional study

**DOI:** 10.1016/S1474-4422(12)70043-1

**Published:** 2012-04

**Authors:** Elisa Majounie, Alan E Renton, Kin Mok, Elise GP Dopper, Adrian Waite, Sara Rollinson, Adriano Chiò, Gabriella Restagno, Nayia Nicolaou, Javier Simon-Sanchez, John C van Swieten, Yevgeniya Abramzon, Janel O Johnson, Michael Sendtner, Roger Pamphlett, Richard W Orrell, Simon Mead, Katie C Sidle, Henry Houlden, Jonathan D Rohrer, Karen E Morrison, Hardev Pall, Kevin Talbot, Olaf Ansorge, The Chromosome 9-ALS/FTD Consortium, The French research network on FTLD/FTLD/ALS, The ITALSGEN Consortium, Dena G Hernandez, Sampath Arepalli, Mario Sabatelli, Gabriele Mora, Massimo Corbo, Fabio Giannini, Andrea Calvo, Elisabet Englund, Giuseppe Borghero, Gian Luca Floris, Anne M Remes, Hannu Laaksovirta, Leo McCluskey, John Q Trojanowski, Vivianna M Van Deerlin, Gerard D Schellenberg, Michael A Nalls, Vivian E Drory, Chin-Song Lu, Tu-Hsueh Yeh, Hiroyuki Ishiura, Yuji Takahashi, Shoji Tsuji, Isabelle Le Ber, Alexis Brice, Carsten Drepper, Nigel Williams, Janine Kirby, Pamela Shaw, John Hardy, Pentti J Tienari, Peter Heutink, Huw R Morris, Stuart Pickering-Brown, Bryan J Traynor

**Affiliations:** a Molecular Genetics Unit, Laboratory of Neurogenetics, National Institute on Aging, National Institutes of Health, Bethesda, MD, USA; b Neuromuscular Diseases Research Unit, Laboratory of Neurogenetics, National Institute on Aging, National Institutes of Health, Bethesda, MD, USA; c Department of Molecular Neuroscience and Reta Lila Weston Laboratories, Institute of Neurology, University College London, Queen Square House, London, UK; d Department of Clinical Neurosciences, Institute of Neurology, University College London, Queen Square House, London, UK; e MRC Prion Unit, Department of Neurodegenerative Disease, Institute of Neurology, University College London, Queen Square House, London, UK; f Department of Molecular Neurosciences and MRC Centre for Neuromuscular Diseases, Institute of Neurology, University College London, Queen Square House, London, UK; g Department of Neurodegenerative Disease, Dementia Research Centre, Institute of Neurology, University College London, Queen Square House, London, UK; h Department of Clinical Genetics, Section of Medical Genomics, and Alzheimer Center, VU University Medical Centre, Amsterdam, Netherlands; i Department of Neurology, Erasmus MC–University Medical Center Rotterdam, Rotterdam, Netherlands; j MRC Centre for Neuropsychiatric Genetics and Genomics, Cardiff University School of Medicine, Cardiff, UK; k Faculty of Human and Medical Sciences, University of Manchester, Manchester, UK; l Department of Neuroscience, University of Turin, Turin, Italy; m Molecular Genetics Unit, Department of Clinical Pathology, Azienda Ospedaliera Ospedale Infantile Regina Margherita Sant Anna, Turin, Italy; n Institute for Clinical Neurobiology, University of Würzburg, Würzburg, Germany; o Department of Pathology, Sydney Medical School, The University of Sydney, NSW, Australia; p Department of Neurology, Institute of Biomedical Research, College of Medical and Dental Sciences, University of Birmingham, Birmingham, UK; q Neurology–University Hospitals Birmingham NHS Foundation Trust, Queen Elizabeth Hospital, Queen Elizabeth Medical Centre, Birmingham, UK; r Nuffield Department of Clinical Neurosciences, John Radcliffe Hospital, University of Oxford, Oxford, UK; s Neurological Institute, Catholic University and ICOMM Association for ALS Research, Rome, Italy; t ALS Center, Salvatore Maugeri Foundation, Milan, Italy; u NeuroMuscular Omnicentre, Niguarda Ca' Granda Hospital, Milan, Italy; v Department of Neurological, Neurosurgical and Behavioural Sciences, Neurology Section, University of Siena, Siena, Italy; w Department of Pathology, Lund University, Regional Laboratories Region Skåne, Lund, Sweden; x Department of Neurology, Azienda Universitaria-Ospedaliera di Cagliari and University of Cagliari, Cagliari, Italy; y Institute of Clinical Medicine, Neurology, University of Oulu and Clinical Research Center, Oulu University Hospital, Oulu, Finland; z Department of Neurology, Helsinki University Central Hospital and Molecular Neurology Programme, Biomedicum, University of Helsinki, Helsinki, Finland; aa Department of Neurology, University of Pennsylvania, Philadelphia, PA, USA; ab Department of Pathology and Laboratory Medicine, University of Pennsylvania, Philadelphia, PA, USA; ac Department of Neurology, Tel-Aviv Sourasky Medical Center, Tel-Aviv, Israel; ad Department of Neurology, Chang Gung Memorial Hospital at Linkou Medical Center and Chang Gung University, Taoyuan, Taiwan; ae Neuroscience Research Center, Chang Gung Memorial Hospital at Linkou Medical Center, Taoyuan, Taiwan; af Department of Neurology, University of Tokyo Hospital, 7–3-1 Hongo, Bunkyo-ku, Tokyo, Japan; ag Université Pierre et Marie Curie-Paris 6, Centre de Recherche de l'Institut du Cerveau et de la Moelle épinière, Paris, France; ah INSERM, U975, Paris, France; ai CNRS, UMR 7225, Paris, France; aj Department of Neuroscience, University of Sheffield, Sheffield, UK; ak Neurology (C4), University Hospital of Wales, Cardiff, UK; al Department of Neurology, Royal Gwent Hospital, Aneurin Bevan Local Health Board, Gwent, UK; am Department of Neurology, Brain Sciences Institute, Johns Hopkins Hospital, Baltimore, MD, USA

## Abstract

**Background:**

We aimed to accurately estimate the frequency of a hexanucleotide repeat expansion in *C9orf72* that has been associated with a large proportion of cases of amyotrophic lateral sclerosis (ALS) and frontotemporal dementia (FTD).

**Methods:**

We screened 4448 patients diagnosed with ALS (El Escorial criteria) and 1425 patients with FTD (Lund-Manchester criteria) from 17 regions worldwide for the GGGGCC hexanucleotide expansion using a repeat-primed PCR assay. We assessed familial disease status on the basis of self-reported family history of similar neurodegenerative diseases at the time of sample collection. We compared haplotype data for 262 patients carrying the expansion with the known Finnish founder risk haplotype across the chromosomal locus. We calculated age-related penetrance using the Kaplan-Meier method with data for 603 individuals with the expansion.

**Findings:**

In patients with sporadic ALS, we identified the repeat expansion in 236 (7·0%) of 3377 white individuals from the USA, Europe, and Australia, two (4·1%) of 49 black individuals from the USA, and six (8·3%) of 72 Hispanic individuals from the USA. The mutation was present in 217 (39·3%) of 552 white individuals with familial ALS from Europe and the USA. 59 (6·0%) of 981 white Europeans with sporadic FTD had the mutation, as did 99 (24·8%) of 400 white Europeans with familial FTD. Data for other ethnic groups were sparse, but we identified one Asian patient with familial ALS (from 20 assessed) and two with familial FTD (from three assessed) who carried the mutation. The mutation was not carried by the three Native Americans or 360 patients from Asia or the Pacific Islands with sporadic ALS who were tested, or by 41 Asian patients with sporadic FTD. All patients with the repeat expansion had (partly or fully) the founder haplotype, suggesting a one-off expansion occurring about 1500 years ago. The pathogenic expansion was non-penetrant in individuals younger than 35 years, 50% penetrant by 58 years, and almost fully penetrant by 80 years.

**Interpretation:**

A common Mendelian genetic lesion in *C9orf72* is implicated in many cases of sporadic and familial ALS and FTD. Testing for this pathogenic expansion should be considered in the management and genetic counselling of patients with these fatal neurodegenerative diseases.

**Funding:**

Full funding sources listed at end of paper (see Acknowledgments).

## Introduction

Amyotrophic lateral sclerosis (ALS) is a fatal neurodegenerative disease characterised by rapidly progressive paralysis and death from respiratory failure, typically within 3 years of symptom onset. The disease is inherited in about 5% of cases, following a clear Mendelian pattern, whereas most cases are classified as sporadic because they seem to arise at random.^1^ Substantial progress has been made in understanding the genetic underpinnings of familial ALS.^2^ By contrast, the causes of sporadic or idiopathic ALS are far less well understood. Mutations in the known familial ALS genes—*SOD1*, *FUS*, and *TDP-43*—occur only rarely in sporadic cases (each accounting for less than 1·0% of cases);^3^, ^4^, ^5^ genome-wide association studies have identified few risk loci, and these have proved difficult to replicate.^6^

Frontotemporal dementia (FTD) is a degenerative disorder of the frontal and anterior temporal lobes, and is a common form of dementia affecting individuals younger than 65 years. The syndrome is characterised clinically by initial behavioural disturbances, followed by cognitive decline leading to dementia and death within a median of 7 years from symptom onset. Akin to ALS and other neurodegenerative diseases, a large proportion (∼60·0%) of these cases are categorised as sporadic, and the causes of this idiopathic form of disease are largely unknown.^7^ A growing consensus suggests that ALS and FTD form part of a continuum of neurological diseases that share a common pathological background, consisting of TAR DNA-binding protein 43 (TDP-43)-positive inclusions within the CNS.^8^

We recently reported that a large hexanucleotide repeat expansion located within the non-coding portion of *C9orf72* is the cause of chromosome 9-linked ALS and FTD.^9^, ^10^ This genetic lesion accounted for a large proportion (∼40·0%) of familial cases of ALS and FTD. The same mutation was present in nearly a quarter of apparently sporadic cases of ALS and FTD in the genetically homogeneous Finnish population, and in 4·1% of sporadic cases of ALS and 3·0% cases of sporadic FTD from the USA. However, these estimates were based on relatively small cohorts drawn from a small number of institutions.

These findings prompted us to aim to estimate the frequency of this *C9orf72* hexanucleotide repeat expansion more accurately, in a large cohort of European and US patients with sporadic ALS and sporadic FTD. We also examined the occurrence of this mutation in diverse non-white populations around the world.

## Methods

### Participants and study design

In this cross-sectional study, we screened 4448 patients diagnosed with ALS and 1425 patients diagnosed with FTD from 17 distinct regions worldwide. The appendix shows ethnic origin and clinical features of the patients. 3860 patients had sporadic ALS, 1022 had sporadic FTD, 588 had familial ALS, and 403 had familial FTD. Data for 401 Finnish patients with ALS, 233 other Europeans with familial ALS, 75 Finnish patients with FTD, 340 Dutch patients with FTD, and 420 English patients with FTD have been published previously.^10^, ^11^, ^12^ All these cohorts were analysed to provide a comprehensive assessment of the global frequency of the expansion.

Patients with ALS were diagnosed according to the El Escorial criteria,^13^ and patients with FTD were diagnosed according to the Lund-Manchester criteria.^14^ We classified patients' disease as familial in nature on the basis of a diagnosis of ALS or FTD in any other family member (irrespective of relationship), as reported at the time of sample collection. We based ethnic and racial classification on self-reports from patients at the time of sample collection. Case numbers listed for European countries and Australia and the Middle East refer to self-reported white individuals from that region. Italian data are from a population-based cohort that had been collected through the Piemonte ALS Registry, an ongoing population-based epidemiological study of ALS based in northwestern Italy.^15^ The remaining cohorts were recruited through medical centres and from repositories in various countries.

We also screened 2585 neurologically healthy control individuals from Australia (213 patients), Finland (478), Germany (309), the Human Gene Diversity Panel (300), mainland Italy (354), Sardinia (87), and the USA (844) for presence of the pathogenic repeat expansion. 1167 of these individuals have been reported elsewhere.^10^ None of the control individuals had been diagnosed with ALS, FTD, dementia, or any other neurodegenerative disease. Ethics committees from the respective institutions approved the study, and written informed consent was obtained from all patients and control individuals.

### Procedures

We used our previously described^10^ repeat-primed PCR assay to screen patients and control individuals for the presence of the chromosome 9p21 GGGGCC hexanucleotide repeat expansion (see appendix for technical details). The assay allows samples to be categorised into those that carry a pathogenic repeat expansion (>30 repeats) and those that carry only wild-type alleles (<20 repeats).

For haplotype analysis, we analysed genome-wide single-nucleotide polymorphism (SNP) data from 262 patients who carried the repeat expansion. We previously reported the identification in the Finnish population of a 42-SNP founder haplotype across the 232 kb block of chromosome 9p21 where the pathogenic hexanucleotide expansion was ultimately established.^16^, ^17^ In this study, we used a custom perl software script to compare unphased sample genotype data with the 42-SNP founder risk haplotype.^16^

We estimated mutation ages for all populations separately with the DMLE+ version 2.3 Bayesian linkage disequilibrium gene mapping package.^18^ Mutation ages were iterated for 10 000 burn-in iterations and a further 10 000 iterations of the maximum-likelihood model. To obtain generalisable estimates of age of the repeat per population, we used median values of binned estimates passing the α threshold of 0·05 per iteration.

### Statistical analysis

We calculated 95% CIs for proportions with the Clopper-Pearson exact method. We estimated penetrance of the GGGGCC hexanucleotide repeat expansion in relation to the patients' age on the basis of data available for 603 mutant-gene carriers with the Kaplan-Meier method using the survival package within R statistical software (version 2.9.0), but substituting patient age at symptom onset for survival time.^19^ We assessed differences between groups with the χ^2^ test for discrete variables such as sex, family history, and site of onset.

### Role of the funding source

The sponsors of the study had no role in study design, data collection, analysis, or interpretation, writing of the report, or in the decision to submit the paper for publication. All authors had full access to all the data in the study and had final responsibility for the decision to submit for publication.

## Results

Table 1 and the appendix show the frequency of the *C9orf72* hexanucleotide repeat expansion in patients diagnosed with sporadic ALS and sporadic FTD from different geographical regions. Data for 289 patients with sporadic ALS and 605 with sporadic FTD have been reported elsewhere.^10^, ^11^, ^12^ The pathogenic expansion was identified in 236 (7·0%) of 3377 white patients from the USA, Europe, the Middle East, and Australia, two (4·1%) of 49 black patients from the USA, and six (8·3%) of 72 Hispanic patients from the USA who were diagnosed with sporadic ALS. The rate of the pathogenic expansion was lower in sporadic FTD: 59 (6·0%) of 981 white patients from Europe carried the mutation. By contrast, the GGGGCC repeat expansion was not present in patients of Native American, Asian, or Pacific Islander origin who had sporadic disease (table 1), although this might reflect the smaller size of the cohorts screened in these populations.

**Table 1:** Frequency of the pathogenic GGGGCC hexanucleotide repeat expansion of *C9orf72* in patients diagnosed with sporadic ALS or sporadic FTD classified by region

		**Sporadic ALS**			**Sporadic FTD**		
		n	Carriers	% (95% CI)	n	Carriers	% (95% CI)
Europe*							
	Finnish	289	61	21·1% (16·5–26·3)	48	9	18·8% (8·9–32·6)
	Swedish	..	..	..	6	0	0% (0·0–45·9)
	English	916	62	6·8% (5·2–8·6)	543	31	5·7% (3·9–8·0)
	German	421	22	5·2% (3·3–7·8)	..	..	..
	Dutch	..	..	..	224	5	2·2% (0·7–5·1)
	French	..	..	..	150	14	9·3% (5·2–15·2)
	Italian	465	19	4·1% (2·5–6·3)	..	..	..
	Sardinian	129	10	7·8% (3·8–13·8)	10	0	0% (0·0–30·8)
	Moldovan	3	0	0% (0·0–70·8)	..	..	..
	Total (Europe)	2223	174	7·8% (6·7–9·0)	981	59	6·0% (4·6–7·7)
USA							
	White	890	48	5·4% (4·0–7·1)	..	..	..
	Hispanic	72	6	8·3% (3·1–17·3)	..	..	..
	Black	49	2	4·1% (0·5–14·0)	..	..	..
	Native American	3	0	0% (0·0–70·8)	..	..	..
	Total (USA)	1014	56	5·5% (4·2–7·1)	..	..	..
Rest of the world							
	Middle Eastern*	1	0	0% (0·0–97·5)	..	..	..
	Indian	31	0	0% (0·0–11·2)	31	0	0% (0·0–11·2)
	Asian	238	0	0% (0·0–1·5)	10	0	0% (0·0–30·8)
	Pacific Islander/Guam	90	0	0% (0·0–4·0)	..	..	..
	Australian*	263	14	5·3% (2·9–8·8)	..	..	..
Overall		3860	244	6·3% (5·6–7·1)	1022	59	5·8% (4·4–7·4)

Data for Finnish (289 with ALS and 48 with FTD), English (333 with FTD), and Dutch (224 with FTD) patients were previously published,^10^, ^11^, ^12^ but are included here to establish global frequencies. ALS=amyotrophic lateral sclerosis. FTD=frontotemporal dementia.

* All self-reported as white.

In addition to sporadic cases, we screened 588 familial cases of ALS and 403 familial cases of FTD for the presence of the *C9orf72* repeat expansion (table 2, appendix). Of these, 345 patients with familial ALS and 230 with familial FTD have been reported elsewhere.^10^, ^11^, ^12^ Overall, 221 (37·6%) of 588 patients with familial ALS and 101 (25·1%) of 403 patients with familial FTD carried the genetic lesion, reinforcing our previous findings that this mutation was responsible for an unparalleled proportion of cases of these diseases.^10^ We identified one Japanese individual diagnosed with familial ALS who carried the hexanucleotide repeat expansion. We also showed that one patient with familial FTD from Lund, Sweden, carried the expansion, suggesting that the chromosome 9p21 genetic lesion might be responsible for the geographical cluster of patients with FTD noted in that region.^20^

**Table 2:** Frequency of the pathogenic GGGGCC hexanucleotide repeat expansion of *C9orf72* in patients diagnosed with familial ALS and familial FTD classified by region

		**Familial ALS**			**Familial FTD**		
		n	Carriers	% (95% CI)	n	Carriers	% (95% CI)
Europe*							
	Finnish	112	52	46·4% (37·0–56·1)	27	13	48·1% (28·7–68·0)
	Swedish	..	..	..	1	1	100·0% (2·5–100·0)
	English	98	45	45·9% (35·8–56·3)	170	28	16·5% (11·2–22·9)
	Irish	1	1	100·0% (2·5–100·0)	..	..	
	German	69	15	21·7% (12·7–33·3)	29	4	13·8% (3·9–31·7)
	Dutch	..	..	..	116	30	25·9% (18·2–34·8)
	French	..	..	..	50	22	44·0% (30·0–58·7)
	Italian	90	34	37·8% (27·8–48·6)	..	..	..
	Sardinian	19	11	57·9% (33·5–79·7)	7	1	14·3% (0·4–57·9)
	Total (Europe)	389	158	40·6% (35·7–45·7)	400	99	24·8% (20·6–29·3)
USA*		163	59	36·2% (28·8–44·1)	..	..	..
Rest of the world							
	Middle Eastern*	2	0	0% (0·0–84·2)	..	..	..
	Israeli*	14	3	21·4% (4·7–50·8)	..	..	..
	Asian	20	1	5·0% (0·1–24·9)	3	2	66·7% (9·4–99·2)
Overall		588	221	37·6% (33·7–41·6)	403	101	25·1% (20·9–29·6)

Data for Finnish (112 with ALS and 27 with FTD), English (87 with FTD), German (41 with ALS), Italian (29 with ALS), US (163 with ALS), and Dutch (116 with FTD) patients were previously published,^10^, ^11^, ^12^ but are included here to establish global frequencies. ALS=amyotrophic lateral sclerosis. FTD=frontotemporal dementia.

* All self-reported as white.

Of 2585 neurologically healthy control samples screened for the *C9orf72* repeat expansion, five (0·2%) were carriers: two were previously reported elderly individuals from Finland,^10^ and the other three were individuals younger than 40 years from Germany and the USA (appendix).

Within Europe, the highest mutation frequency was noted in the Finnish population (21·1% of patients with sporadic ALS and 18·8% of patients with sporadic FTD).^10^ About 6% of patients with sporadic ALS from Germany and England carried the expansion, whereas Italian patients with ALS had a lower rate (4·1%). 7·8% of patients with sporadic ALS from the genetically isolated island population of Sardinia had the mutation and the Dutch population had the lowest detected rate observed in European countries (2·2% of sporadic cases of FTD). White populations from Australia and the USA had an intermediate rate, with about 5·0% of patients with sporadic ALS carrying the pathogenic repeat expansion, perhaps because of the population and immigration histories of these countries.

**Figure 1:**
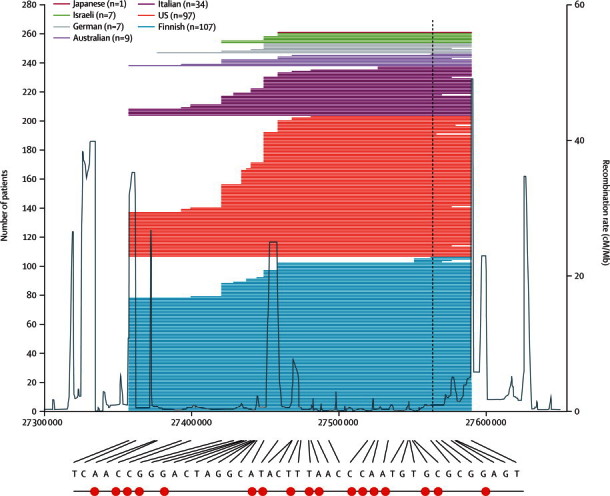
Finnish risk haplotypes across the chromosome 9p21 region in 262 patients with amyotrophic lateral sclerosis and the *C9orf72* mutation The previously identified Finnish risk haplotype is shown below the graph (27 357 278–27 589 746 bp; NCBI build 36; 42 single nucleotide polymorphisms [SNPs]).^16^ Underneath the haplotype is a binary representation of the same data, with red circles at SNP positions where the haplotype has the less common allele at that site. In the graph, individual patients are shown as horizontal lines showing the extent to which they share the risk haplotype. The vertical black dashed line shows the location of the *C9orf72* hexanucleotide repeat expansion. Recombination rates (centimorgans per megabase [cM/Mb]) from phase 2 Centre d'Etude du Polymorphisme Humain (CEPH) samples of HapMap are shown with a grey line.

Haplotype analysis suggested that every patient carrying the pathogenic GGGGCC repeat expansion also shared the Finnish founder risk haplotype, at least in part (figure 1). Furthermore, patients with sporadic and familial disease carried the same founder risk haplotype. These findings suggest that the pathogenic hexanucleotide repeat expansion in *C9orf72* might have occurred on one occasion in human history and subsequently disseminated throughout these populations. Analysis of haplotype sharing between these cases estimated the age of *C9orf72* repeat expansion to be about 1500 years old (representing a median of 100·5 generations [IQR 57·6–127·6], assuming a generation is 15 years old).

**Figure 2:**
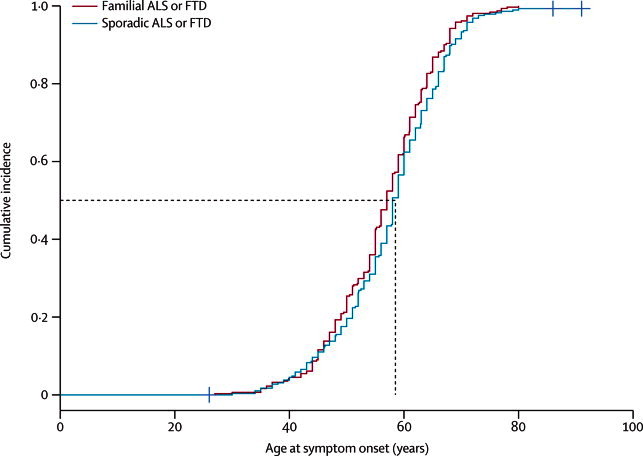
Age-related penetrance of the GGGGCC hexanucleotide repeat expansion in *C9orf72* Kaplan-Meier analysis of 603 mutant-gene carriers (212 patients with familial amyotrophic lateral sclerosis, 234 with sporadic amyotrophic lateral sclerosis, 99 with familial frontotemporal dementia, 53 with sporadic frontotemporal dementia, and five neurologically healthy controls). Age-related penetrance (ie, the proportion of mutant-gene carriers with manifestations of the disease by a given age) rose steadily, from 10% in patients younger than 45 years to almost 100% by the age of 80 years. The dotted lines shows the age at which 50% of the cohort developed symptoms. Vertical blue lines show censored events.

In analysis of age-related penetrance (figure 2), the pathogenic expansion was non-penetrant in carriers who were younger than 35 years of age, increasing to 50% penetrance by 58 years, and to almost full penetrance by 80 years. We noted no difference between disease penetrance according to familial status, ALS or FTD diagnosis, sex, or age of symptom onset in patients with ALS or FTD (appendix).

Table 3 shows clinical details of patients carrying the hexanucleotide repeat expansion. Patients with ALS and the pathogenic repeat expansion were more likely to be female (p=0·0008), have a family history of disease (p<0·0001), and to have bulbar-onset disease (p=0·0011) than were patients who did not carry the expansion. Patients with FTD carrying the repeat expansion were also more likely to have a family history of disease (p<0·0001) and to present with behavioural variant FTD (p<0·0001).

**Table 3:** Demographic and clinical features of patients classified by diagnosis and by carrier status for the GGGGCC hexanucleotide repeat expansion in *C9orf72*

		**Amyotrophic lateral sclerosis**		**Frontotemporal dementia**	
		With expansion (n=465)*	Without expansion (n=3983)†	With expansion (n=160)‡	Without expansion (n=1265)§
Mean age at onset (range; SD)		56·8 (27·0–80·0; 9·1)	58·7 (4·0–93·0; 12·8)	57·5 (30·0–76·3; 8·3)	60·0 (23·0–87·0; 8·8)
Sex, male		232 (50·1%)	2251 (58·4%)	87 (54·4%)	683 (55·4%)
Positive family history		221 (47·5%)	367 (9·2%)	101 (63·1%)	302 (23·9%)
Presentation					
	Bulbar	139 (33·1%)	933 (26·0%)	..	..
	Limb	281 (66·9%)	2655 (74·0%)	..	..
	Behavioural variant	..	..	106 (85·5%)	685 (65·6%)
	Progressive non-fluent aphasia	..	..	11 (8·9%)	165 (15·8%)
	Semantic dementia	..	..	7 (5·6%)	195 (18·6%)

Data are mean (range; SD) or n (%).

* Data not available for age at onset for 19 patients and site of onset for 45 patients.

† Data not available for age at onset for 305 patients, sex for 130 patients, and site of onset for 395 patients.

‡ Data not available for age at onset for eight patients and site of onset for 36 patients.

§ Data not available for age at onset for 71 patients, sex for32 patients, and site of onset for 220 patients.

## Discussion

Our data show that the *C9orf72* hexanucleotide repeat expansion is the most frequent cause of sporadic ALS and sporadic FTD identified thus far, accounting for about 5·0–7·0% of cases in white Europeans, Americans, and Australians in our large cohort. These frequency rates were slightly higher than were estimates from smaller cohorts obtained at one institution.^9^ Before identification of the genetic lesion underlying chromosome 9-linked ALS and FTD, mutations in the *SOD1* gene were the most common known genetic cause of sporadic ALS (accounting for 0·7% of cases in a population-based cohort),^3^ whereas mutations in the *PGRN* gene were the most common known cause of sporadic FTD (3·0–4·0% in clinic referral series).^21^ The high frequency of the pathogenic expansion in our cohort is consistent with previous genome-wide association studies that identified the association signal on chromosome 9p21 as the only replicable locus in the sporadic form of ALS and FTD.^16^, ^22^, ^23^, ^24^ Our findings confirm the importance of genetics in the pathogenesis of the idiopathic form of these neurodegenerative diseases.

Our haplotype data suggest that the pathogenic GGGGCC hexanucleotide repeat expansion in *C9orf72* arose from a one-off mutation event^16^, ^17^ that occurred about 1500 years ago. The geographical distribution of the mutation suggests that the mutation appeared in northern Europe and spread from there. Alternatively, the high frequencies in Finland and other isolated populations could be explained by the history of these communities. Finland and Sardinia are comparatively isolated regions, and have genetically homogeneous populations that originated from a small number of founders.^25^ Genetic drift has had a large influence on allele frequencies in these populations and could explain the high occurrence of the mutation in these geographical isolates.

Recognition that all patients carrying the *C9orf72* repeat expansion share a common ancestor has important implications for the interpretation of global frequency data for this mutation. Although the hexanucleotide repeat expansion is common in white Europeans, it is also present in black and Hispanic populations in the USA and individuals from Israel. This finding probably reflects the scale and nature of past human migration and intermarriage between ethnic groups. Similarly, the relative absence of the pathogenic hexanucleotide repeat in India, Asia, and the Pacific Islands might be explained by the greater physical distances of these regions from Europe, and the consequent lack of admixture between populations. Notably, the one Japanese patient who we identified as a carrier of the *C9orf72* expansion carried the Finnish risk haplotype, reinforcing the notion that the expansion occurred on one occasion in the past.

The sharing of a common risk haplotype in the *C9orf72* region of chromosome 9p21 in patients with sporadic and familial ALS suggests that these apparently sporadic cases are actually cryptically related familial cases. This scenario might have occurred for several reasons, including unfamiliarity with the pedigree on the part of the patient or neurologist or because previous generations might have died at a young age before onset of neurological symptoms. The median age at onset in patients with the expansion was 57 years, and life expectancy in the USA began to exceed this point only in the early 1940s.^26^ Furthermore, the incomplete penetrance of the mutation, in which not all individuals carrying the expansion manifest a clinical phenotype, might be a contributing factor in apparently sporadic disease. Indeed, we have reported symptom onset in the ninth decade of life in patients carrying the expansion and also encountered two elderly, neurologically healthy individuals with the expansion. Thus, the penetrance of this mutation seems to be complete only at a late stage of life, which is an observation of particular relevance for genetic counselling of healthy individuals carrying the expansion. The molecular biological substrate underlying this variability in age at onset is unclear: it might be driven by differences in expansion lengths between patients, by age-related methylation across the locus, or by genetic factors elsewhere in the genome.

We compared our results with those of previous studies that reported the frequency of the *C9orf72* hexanucleotide repeat expansion in the pathogenesis of ALS and FTD (panel). Data were available from seven studies (appendix). Our study screened one of the largest cohorts of cases of ALS and FTD assessed to date, and also provides an initial report of the frequency of the pathogenic repeat expansion in non-white patients, a detailed examination of the haplotype across the locus, and an initial estimate of age-related disease penetrance in a large group of individuals carrying the expansion.

Panel **Research in context**

**Systematic review**

We searched Medline up to December, 2011, without language restrictions for relevant publications and selected studies that reported the GGGGCC hexanucleotide repeat expansion in *C9orf72* in pathogenesis of amyotrophic lateral sclerosis (ALS) or frontotemporal dementia (FTD). On the basis of these criteria, seven studies were identified for further assessment (appendix). The number of patients screened for the pathogenic repeat expansion and the phenotype and ethnic origin reported by these studies are summarised in the appendix.

**Interpretation**

We report the frequency of the *C9orf72* repeat expansion in a large cohort of patients with sporadic ALS and sporadic FTD. We also screened a large number of non-white patients for the expansion, and present frequency data for the mutation in these populations. We confirmed that the *C9orf72* repeat expansion explains a substantial proportion of sporadic ALS (∼7·0%) and sporadic FTD (∼6·0%) cases in white populations. We also noted that patients with sporadic and familial disease carrying the expansion share a founder risk haplotype, suggesting that these patients have a common ancestor and that the original mutational event that led to the repeat expansion occurred only once in the past. We provide initial estimates of age-related penetrance, showing that 50% of carriers manifest disease by 58 years of age, and that the mutation is fully penetrant by 80 years of age.

Our data have implications for the clinical care of patients diagnosed with ALS and FTD. The clinical standard of care is to offer genetic testing to patients reporting a family history of ALS or FTD,^27^ and to reassure patients classified as having sporadic disease that their relatives are not at increased risk of neurodegeneration. On the basis of an analysis of 191 Irish patients with ALS, Byrne and colleagues^28^ suggested that genetic testing for the *C9orf72* repeat expansion is unnecessary in affected individuals without a family history of disease or substantial cognitive impairment. By contrast, we believe that genetic testing is a valuable technique for accurate diagnosis of the two disorders and in the decision-making process for patients and their families. The discrepancy between these two views might stem from differences in how sporadic and familial disease were defined in the two studies. Accumulation of sufficient data is an important step towards answering this key question for management of patients. In view of the large number of patients who carry the repeat expansion, investigators and clinicians should at least consider a focused debate on this issue.

Our paper has some limitations. First, the number of patients from some geographical regions was small and the mutational frequencies might change for those ethnic groups as additional patients are screened. Nevertheless, our data for more than 5000 patients with ALS or FTD provide a reasonable estimation of *C9orf72* global frequency. Second, although we have examined the chromosome 9p21 haplotype in a large and diverse cohort of individuals carrying the pathogenic expansion, additional testing of carriers might reveal other haplotypes, thereby indicating that the expansion arose on more than one occasion. Nevertheless, our data suggest that most expansion carriers share a common ancestor.^16^, ^17^ Third, we generated age-related penetrance estimates on the basis of data from retrospective cohorts, which potentially leads to overestimation of penetrance. Additional prospective studies examining family kindreds are necessary to confirm these estimates. Finally, case classification as familial or sporadic was done on the basis of clinical questioning at sample collection. The level of scrutiny might have varied between centres and countries, but re-collection of this information for existing cohorts was not feasible.

## Web Extra Material

Supplementary webappendix
