# Supplementary material for: Frequency of the C9orf72 hexanucleotide repeat expansion in patients with amyotrophic lateral sclerosis and frontotemporal dementia: a cross-sectional study
Source: Lancet Neurol. 2012 Apr;11(4):323–30. doi: 10.1016/S1474-4422(12)70043-1 (PMC3322422; doi:10.1016/S1474-4422(12)70043-1)
Supplement: Supplementary webappendix [file mmc1.pdf]

THE LANCET Neurology

Supplementary webappendix

This webappendix formed part of the original submission and has been peer reviewed. We post it as supplied by the authors.

Supplement to: Majounie E, Renton AE, Mok K, et al. Frequency of the *C9orf72* hexanucleotide repeat expansion in patients with amyotrophic lateral sclerosis and frontotemporal dementia: a cross-sectional study. *Lancet Neurol* 2012; published online March 9. DOI:10.1016/S1474-4422(12)70043-1.

Supplementary webappendix

Supplement to: Majounie E, Renton AE, Mok K, et al. Frequency of the *C9ORF72* hexanucleotide repeat expansion in ALS and FTD in diverse populations: a cross-sectional study. *Lancet Neurol*

The Chromosome 9-ALS/FTD Consortium
Gary Adamson BSc[1], Antony J. Bayer MD[2], John Beck BSc[1], Janis Bennion Callister PhD[3], Derek J Blake MD[4], Sergiu C. Blumen MD[5], John Collinge MD[1], Travis Dunckley PhD[6], John Ealing MD[7], Simon East DPhil[8], Lauren Elman MD[9], Alexander Gerhard MD[10], Rita J. Guerreiro PhD[11,] Katrina Gwinn MD[12], Nicola Halliwell BSc[3], Hisham H. Hamdalla MD[13], Christopher Hewitt[14], Paul Ince MD[14], Sibylle Jablonka MD[15], Christopher James MD[16], Louisa Kent MD[17], Jonathan Cooper Knock MD[14], Timothy Lynch MD[18], Colin Mahoney MD[1], David Mann PhD[10], James Neal MD[19], David Norris MD[20], Sean O'Dowd MD[18], Anna Richardson MD[10], Martin Rossor MD[21], Jeffrey Rothstein MD[22], Sonja W. Scholz MD[22], Julie Snowden PhD[10], Dietrich A. Stephan[23,24], Greg Toulson BSc[3], Martin R Turner[17], Jason D. Warren MD[1], Kate Young BSc[3], Yi-Hsin Weng[25,26], Hung-Chou Kuo[25,26], Szu-Chia Lai[25,26], Chia-Ling Huang.[27]

Affiliations
[1] MRC Prion Unit, Department of Neurodegenerative Disease, Institute of Neurology, University College London, Queen Square House, London WC1N 3BG, UK
[2] Section of Geriatric Medicine, School of Medicine, Cardiff University, Cardiff, CF14 4XN, UK
[3] Faculty of Human and Medical Sciences, University of Manchester, Manchester M13 9PT, UK
[4] MRC Centre for Neuropsychiatric Genetics and Genomics, Cardiff University School of Medicine, Cardiff CF14 4XN, UK
[5] Department of Neurology, Hillel-Yaffe Medical Center, Hadera, 38100 Israel.
[6] Neurogenomics Division, Translational Genomics Institute (TGEN), Phoenix, AZ 85004, USA
[7] Department of Neurology, Hope Hospital, Stott Lane, Greater Manchester M6 8HD, UK
[8] Neuropathology, Nuffield Department of Clinical Neurosciences, John Radcliffe Hospital, University of Oxford, Oxford OX3 9DU, UK
[9] Department of Neurology, University of Pennsylvania, Philadelphia, PA 19104, USA
[10] Neurodegeneration and Mental Health Research Group, School of Community Based Medicine, The University of Manchester, Manchester M20 3LJ, UK
[11] Department of Molecular Neuroscience and Reta Lila Weston Laboratories, Institute of Neurology, University College London, Queen Square House, London WC1N 3BG, UK
[12] National Institutes of Neurological Disorders and Stroke, NIH, Bethesda, MD 20852, USA
[13] Department of Neurology Hull Royal Infirmary, Kingston upon Hull HU3 2JZ, UK
[14] Department of Neuroscience, University of Sheffield, Sheffield S10 2HQ, UK
[15] Institute for Clinical Neurobiology, University of Würzburg, D-97078 Würzburg, Germany
[16] Withybush Hospital, Hywel Dda Health Board, Haverfordwest, Pembrokeshire SA61 2PZ, UK
[17] Nuffield Department of Clinical Neurology, John Radcliffe Hospital, Oxford OX3 9DU, UK
[18] The Dublin Neurological Institute, Dublin 7, Ireland.
[19] Department of Pathology, Cardiff University School of Medicine, Cardiff CF14 4XN, UK
[20] MRC Cognition and Brain Sciences Unit, Cambridge CB27EF, UK
[21] Dementia Research Centre, Institute of Neurology, University College London, Queen Square House, London WC1N 3BG, UK
[22] Department of Neurology, Brain Sciences Institute, Johns Hopkins University, Baltimore, MD 21287, USA
[23] Navigenics, Foster City, CA 94404, USA
[24] IGNITE Institute for Individualized Health, Fairfax, VA 22042, USA
[25] Department of Neurology, Chang Gung Memorial Hospital at Linkou Medical Center and Chang Gung University, Taoyuan, Taiwan
[26] Neuroscience Research Center, Chang Gung Memorial Hospital at Linkou Medical Center, Taoyuan, Taiwan
[27] Department of Neurology, Saint Paul's Hospital, Taoyuan, Taiwan

The ITALSGEN Consortium
Cristina Moglia MD[1], Stefania Cammarosano MD[1], Antonio Canosa MD[1], Sara Gallo MD[1], Maura Brunetti BS[2], Irene Ossola BS[2], Kalliopi Marinou MD[3], Laura Papetti PharmD[3], Fabrizio Pisano MD[3], Giuseppe Lauria Pinter MD[4], Amelia Conte MD[5], Marco Luigetti MD[5], Marcella Zollino MD[6], Serena Lattante BS[6], Giuseppe Marangi MD[6], Vincenzo la Bella MD[7], Rossella Spataro MD[7], Tiziana Colletti MD[7], Stefania Battistini MD[8], Claudia Ricci MD[8], Claudia Caponnetto MD[9], Gianluigi Mancardi MD[9], Paola Mandich MD[9], Fabrizio Salvi MD[10], Ilaria Bartolomei MD[10], Jessica Mandrioli MD[11], Patrizia Sola MD[11], Christian Lunetta MD[12], Silvana Penco[13], Maria Rosaria Monsurrò MD[14], Gioacchino Tedeschi MD[14], Francesca Luisa Conforti PhD[15], Antonio Gambardella MD[15], Aldo Quattrone MD[15], Paolo Volanti MD[16], Gianluca Floris MD[17], Antonino Cannas MD[17], Valeria Piras MD[17], Francesco Marrosu MD[17], Maria Giovanna Marrosu MD[18], Maria Rita Murru BS[18], Maura Pugliatti MD[19], Leslie D. Parish MD[19], Alessandra Sotgiu MD[20], Giuliana Solinas PhD[20], Lucia Ulgheri PhD[20], Anna Ticca MD[21], Isabella Simone MD[22], and Giancarlo Logroscino MD[22].

Affiliations
[1] Department of Neuroscience, University of Turin, Turin, Italy
[2] Laboratory of Molecular Genetics, ASO OIRM-Sant'Anna, Turin, Italy
[3] Salvatore Maugeri Foundation, IRCCS, Scientific Institute of Milan, Milan, Italy
[4] Department of Neurology, National Institute of Neurology 'Carlo Besta', Milan, Italy
[5] Neurological Institute, Catholic University and I.CO.M.M. Association for ALS Research, Rome, Italy
[6] Molecular Genetics Laboratory, Catholic University of Rome, Rome, Italy
[7] ALS Clinical Research Center, Bio.Ne.C., University of Palermo, Palermo, Italy
[8] Department of Neuroscience, Neurology Section, University of Siena, Siena, Italy
[9] Department of Neuroscience, Ophthalmology and Genetics, University of Genoa, Genoa Italy
[10] Center for Diagnosis and Cure of Rare Diseases, Department of Neurology, Bellaria Hospital, Bologna, Italy
[11] Department of Neuroscience, S. Agostino- Estense Hospital, and University of Modena, Modena, Italy
[12] NeuroMuscular Omnicenter, Serena Foundation, Milan
[13] Department of Laboratory Medicine, Medical Genetics, Niguarda Ca' Granda Hospital, Milan, Italy
[14] Department of Neurological Sciences, Second University of Naples, Naples, Italy
[15] Institute of Neurological Sciences, National Research Council, Mangone, Cosenza, Italy
[16] Salvatore Maugeri Foundation, IRCCS, Scientific Institute of Mistretta, Mistretta, Italy
[17] Azienda Universitaria-Ospedaliera di Cagliari, and University of Cagliari, Cagliari, Italy
[18] Centro Sclerosi Multipla, Ospedale Binaghi, Università di Cagliari, Cagliari, Italy
[19] Department of Neuroscience, University of Sassari, Italy
[20] Department of Biomedical Science, University of Sassari, Sassari, Italy,
[21] AO San Francesco, Nuoro, Italy
[22] Department of Neuroscience, University of Bari, Bari, Italy

The French research network on FTLD/FTLD-ALS
Agnès Camuzat[1], Léna Entraingues[1], Guillot-Noël[1], Patrice Verpillat[1], Frédéric Blanc[2], William Camu[3], Françoise Clerget-Darpoux[4], Philippe Corcia[5], Philippe Couratier[6], Mira Didic[7], Bruno Dubois[8], Charles Duyckaerts[9], Eric Guedj[10], Véronique Golfier[11], Marie-Odile Habert[12], Didier Hannequin[13], Lucette Lacomblez[14], Vincent Meininger[14], François Salachas[14], Richard Levy[15], Bernard-François Michel[16], Florence Pasquier[17], Michèle Puel[18], Catherine Thomas-Anterion[19], François Sellal[20], and Martine Vercelletto[21].

Affiliations
[1] CRicm, UPMC, Inserm UMR_S975/CNRS UMR 7225, Paris, France
[2]Département de Neurologie, Hôpitaux Civils, Strasbourg, France
[3]Service de Neurologie, CHU Gui de Chauliac, Montpellier, France
[4]Inserm U535, Hôpital Paul Brousse, Villejuif, France
[5]Service de Neurologie et de Neurophysiologie Clinique CHU Bretonneau, Tours, France
[6]EA3174, CHU Dupuytren, Limoges, France
[7]Service de Neurologie et Neuropsychologie, CHU La Timone, Marseille, France
[8]Institut de la Mémoire et de la Maladie d'Alzheimer, Hôpital de la Salpêtrière, Paris, France
[9]Laboratoire de Neuropathologie Escourolle, Hôpital de la Salpêtrière, Paris, France
[10]Service de Médecine Nucléaire, CHU La Timone, Marseille, France
[11]Service de Neurologie, CHU, Rennes, France

[12]Service de Médecine Nucléaire, Hôpital de la Salpêtrière, Paris, France
[13]Service de Neurologie, Rouen University Hospital, France
[14]Département de Neurologie, Hôpital de la Salpêtrière, Paris, France
[15]Service de Neurologie, Hopital Saint Antoine, Paris, France
[16]CNRS UMR 6149, Hôpital Sainte-Marguerite, Marseille, France
[17]Clinique Neurologique, CHU Roger Salengro, Lille, France
[18]Inserm U825, CHU Purpan, Toulouse, France
[19]Service de Neurologie, CHU Bellevue, Saint-Etienne, France
[20]Département de Neurologie et INSERM U692, Hôpitaux Civils, Strasbourg, France
[21]Clinique neurologique, CHU Laennec, Nantes, France

Supplementary methods

Repeat-primed PCR

Briefly, 100ng of genomic DNA were used as template in a final volume of 28ul containing 14ul of FastStart PCR Master Mix (Roche Applied Science, Indianapolis, IN, USA), and a final concentration of 0·18mM 7-deaza-dGTP (New England Biolabs Inc., Ipswich, MA, USA), 1x Q-Solution (Qiagen Inc., Valencia, CA, USA), 7% DMSO (Qiagen), 0·9mM $MgCl_2$ (Qiagen), 0·7uM reverse primer consisting of ~ four GGGGCC repeats with an anchor tail (TACGCATCCCAGTTTGAGACGGGGGCCGGGGCCGGGGCCGGGG), 1·4uM 6FAM-fluorescent labeled forward primer located 280bp telomeric to the repeat sequence (AGTCGCTAGAGGCGAAAGC), and 1·4uM anchor primer corresponding to the anchor tail of the reverse primer (TACGCATCCCAGTTTGAGACG).[1,2] A touchdown PCR cycling program was used where the annealing temperature was gradually lowered from 70°C to 56°C in 2°C increments with a 3-minute extension time for each cycle. Fragment length analysis was performed on an ABI 3730xl genetic analyzer (Applied Biosystems Inc., Foster City, CA, USA), and data analyzed using GeneScan software (version 4, ABI). Repeat expansions produce a characteristic sawtooth pattern with a 6-bp periodicity when fragment lengths are analyzed on a capillary-based sequencer.[3]

Supplementary Table 1. Demographics and clinical features of patients screened for the GGGGCC hexanucleotide repeat expansion of the *C9ORF72* gene classified by diagnosis and familial status. (A) Demographic and clinical features of patients diagnosed with sporadic ALS screened for the GGGGCC hexanucleotide repeat expansion in the *C9ORF72* gene.

				Site of symptom onset	
**Origin**	**N**	**Age at onset (range)**	**Male (%)**	**Bulbar-onset (%)**	**Spinal-onset (%)**
**Europe:**					
Finnish	289	58·2 (30·0-85·0)	149 (51·6%)	74 (31·2%)	163 (68·8%)
English	916	60·1 (16·0-86·0)	534 (58·9%)	237 (29·9%)	555 (70·1%)
German	421	57·3 (16·0 - 85·0)	263 (62·5%)	87 (20·7%)	334 (79·3%)
Italian	465	61·7 (20·5 - 87·3)	258 (55·5%)	127 (27·3%)	338 (72·7%)
Sardinian	129	59·7 (27·0 - 82·0)	80 (62·0%)	27 (20·9%)	102 (79·1%)
Moldovan	3	MD	3 (100·0%)	MD	MD
**United States:**					
White	890	56·5 (19·0 - 93·0)	514 (57·8%)	179 (21·2%)	666 (78·8%)
Hispanic	72	58·8 (15·0 - 83·0)	44 (61·1%)	37 (52·9%)	33 (47·1%)
African American	49	55·2 (14·0 - 82·0)	20 (40·8%)	17 (34·7%)	32 (65·3%)
Native American	3	61·0 (56·0 - 66·0)	1 (33·3%)	0 (0·0%)	3 (100·0%)
**Global:**					
Middle Eastern	1	73·0	1 (100·0%)	0 (0·0%)	1 (100·0%)
Indian	31	MD	MD	MD	MD
Asian	238	59·2 (24·0 - 89·0)	148 (62·2%)	61 (28·6%)	152 (71·4%)
Pacific Islander/Guam	90	53·7 (43·0 - 70·0)	3 (100·0%)	2 (66·7%)	1 (33·3%)
Australian	263	64·0 (25·0 - 90·0)	171 (65·0%)	78 (29·7%)	185 (70·3%)

MD = missing data; data was not available for age at onset (n = 12 Finnish patients; n = 134 English; n = 3 Moldovan; n = 1 US white; n = 31 Indian; n = 3 Asians; n = 87 Pacific Islanders), for gender (n = 10 English patients; n = 31 Indian; n = 87 Pacific Islanders), and for site of onset (n = 52 Finnish patients; n = 124 English; n = 3 Moldovan, n = 34 US white; n = 2 US Hispanic; n = 31 Indian; n = 25 Asians; n = 87 Pacific Islanders).

Supplementary Table 1 (B). Demographic and clinical features of patients diagnosed with familial ALS screened for the GGGGCC hexanucleotide repeat expansion in the *C9ORF72* gene.

Origin	N	Age at onset (range)	Male (%)	Site of symptom onset	
				Bulbar-onset (%)	Spinal-onset (%)
**Europe:**					
Finnish	112	52·8 (18·0 - 81·0)	49 (43·8%)	19 (20·7%)	73 (79·3%)
English	98	56·5 (27·0 - 88·0)	47 (48·5%)	25 (30·1%)	58 (69·9%)
Irish	1	45	1 (100·0%)	0 (0·0%)	1 (100·0%)
German	69	52·0 (23·0 - 90·0)	35 (50·7%)	11 (37·9%)	18 (62·1%)
Italian	90	58·9 (18·0 - 79·0)	49 (54·4%)	32 (35·6%)	58 (64·4%)
Sardinian	19	58·7 (33·0 - 78·0)	10 (52·6%)	7 (36·8%)	12 (63·2%)
**United States:**					
White	163	54·8 (15·0 - 80·0)	87 (53·3%)	40 (25·2%)	119 (74·8%)
**Global:**					
Middle Eastern	2	32·5 (29·0 - 36·0)	1 (50·0%)	0 (0·0%)	1 (100·0%)
Israeli	14	49·4 (22·0 - 74·0)	5 (35·7%)	2 (14·3%)	12 (85·7%)
Asian	20	47·3 (4·0 - 77·0)	11 (57·9%)	4 (22·2%)	14 (77·8%)

Data was not available for age at onset (n = 17 Finnish patients; n = 10 English; n = 1 US White; n = 1 Asian), for gender (n = 1 English patient; n = 1 Asian patient), and for site of onset (n = 20 Finnish patients; n = 15 English; n = 40 Germans; n = 4 US White; n = 1 Middle Eastern; n = 2 Asians).

Supplementary Table 1 (C). Demographic and clinical features of patients diagnosed with sporadic FTD screened for the GGGGCC hexanucleotide repeat expansion in the *C9ORF72* gene.

Origin	N	Age at onset (range)	Male (%)	Type of FTD at presentation		
				**Behavioural**	**PNFA**	**Semantic dementia**
**Europe***:*						
Finnish	48	58·0 (38·0 - 73·0)	19 (39·6%)	30 (62·5%)	14 (29·2%)	4 (8·3%)
Swedish	6	60·2 (32·0 - 75·0)	4 (66·7%)	5 (100·0%)	0 (0·0%)	0 (0·0%)
English	543	60·8 (23·0 - 87·0)	306 (56·5%)	284 (57·0%)	102 (20·5%)	112 (22·5%)
Dutch	224	57·6 (28·6 - 76·0)	111 (49·6%)	156 (69·6%)	19 (8·5%)	49 (21·9%)
French	150	62·4 (40·0 - 79·0)	92 (61·3%)	93 (97·8%)	1 (1·1%)	1 (1·1%)
Sardinian	10	68·4 (59·0 - 79·0)	6 (60·0%)	7 (70·0%)	3 (30·0%)	0 (0·0%)
**Global:**						
Indian	31	MD	MD	MD	MD	MD
Asian	10	57·8 (44·0 - 80·0)	3 (30·0%)	9 (81·8%)	1 (9·2%)	0 (0·0%)

PNFA, progressive non-fluent aphasia; MD = missing data; Data was not available for age at onset (n = 16 English patients; n = 14 French; n = 31 Indians; n = 2 Sardinians), for gender (n = 1 English patient; n = 31 Indians), and for type of FTD at onset (n = 1 Swedish patient; n = 45 English patients; n = 55 French; n = 31 Indian patients).

Supplementary Table 1 (D). Demographic and clinical features of patients diagnosed with familial FTD screened for the GGGGCC hexanucleotide repeat expansion in the *C9ORF72* gene.

				Type of FTD at presentation		
**Origin**	**N**	**Age at onset (range)**	**Male (%)**	**Behavioural**	**PNFA**	**Semantic dementia**
**Europe:**						
Finnish	27	59·0 (46·0 - 79·0)	15 (55·6%)	18 (66·7%)	6 (22·2%)	3 (11·1%)
Swedish	1	68·0	0 (0·0%)	1 (100·0%)	0 (0·0%)	0 (0·0%)
English	170	58·1 (39·0 - 83·0)	95 (55·9%)	50 (69·4%)	11 (15·3%)	11 (15·3%)
German	29	MD	MD	MD	MD	MD
Dutch	116	58·6 (36·2 - 76·3)	61 (52·6%)	87 (75·0%)	13 (11·2%)	16 (13·9%)
French	50	59·0 (30·0 - 75·0)	33 (66·0%)	24 (100·0%)	0 (0·0%)	0 (0·0%)
Sardinian	7	63·3 (49·0 - 74·0)	5 (71·4%)	6 (85·7%)	1 (14·3%)	0 (0·0%)
**Global:**						
Asian	3	51·7 (45·0 - 56·0)	1 (33·3%)	3 (100·0%)	0 (0·0%)	0 (0·0%)

PNFA, progressive non-fluent aphasia; MD = missing data; Data was not available for age at onset (n = 6 English patients; n = 29 Germans; n = 10 French), for gender (n = 29 Germans), and for type of FTD at onset (n = 98 English patients; n = 29 Germans; n = 26 French).

Supplementary Table 2. Demographics and clinical features of neurologically normal individuals carrying the GGGGCC hexanucleotide repeat expansion in the *C9ORF72* gene.

	Age at reported collection	Gender	Race	Ethnicity	Country of origin	Comment
**Vantaa1**	85-90 years	M	W	Non-Hispanic	Finland	Deceased without personal history of neurological disease
**Vantaa2**	90-95 years	F	W	Non-Hispanic	Finland	Deceased without personal history of neurological disease
**German1**	35-40 years	F	W	Non-Hispanic	German	No reported history of neurological disease at time of sample collection
**German2**	25-30 years	F	W	Non-Hispanic	German	No reported history of neurological disease at time of sample collection
**ND15567**	36	F	W	Non-Hispanic	US	No reported personal or family history of neurological diseases

Additional details are available for ND15567 at www.coriell.org; To protect privacy, the sample IDs of the other samples has been modified, and their age at collection has been provided as a 5 year range.

Supplementary Table 3. Systematic review of relevant publications that reported the GGGGCC hexanucleotide repeat expansion of the *C9ORF72* gene relevant to the pathogenesis of ALS or FTD.

Study	Reference#	N	Mutation frequency (%)	Phenotype	Race
Renton et al	3	112	52 (46·4%)	Familial ALS	Finnish
Renton et al	3	290	61 (21·0%)	Sporadic ALS	Finnish
Renton et al	3	75	22 (29·3%)	FTD	Finnish
Renton et al	3	268	102 (38·1%)	Familial ALS	Outbred European-ancestry
DeJesus-Hernandez et al	4	34	8 (23·5)	Familial ALS	US
DeJesus-Hernandez et al	4	195	8 (4·1)	Sporadic ALS	US
DeJesus-Hernandez et al	4	171	20 (11·7%)	Familial FTD	US
DeJesus-Hernandez et al	4	203	6 (3·0)	Sporadic FTD	US
Byrne et al	5	49	20 (40·8%)	Familial ALS	Irish
Byrne et al	5	386	19 (4·9%)	Sporadic ALS	Irish
Simon-Sanchez et al	6	129	37 (28·7%)	Familial FTD	Dutch
Simon-Sanchez et al	6	224	5 (2·2%)	Sporadic FTD	Dutch
Snowden et al	7	398	32 (8·0%)	FTD	English
Stewart et al	8	62	17 (27·4%)	Familial ALS	US
Stewart et al	8	169	6 (3·6%)	Sporadic ALS	US
Gijselinck et al	9	15	7 (46·7%)	Familial ALS	Belgian
Gijselinck et al	9	122	6 (4·9%)	Sporadic ALS	Belgian
Gijselinck et al	9	82	18 (22·0%)	Familial FTD	Belgian
Gijselinck et al	9	246	10 (4·1%)	Sporadic FTD	Belgian

Supplementary Figure 1. Geographical distribution of the pathogenic GGGGCC hexanucleotide repeat expansion of the *C9ORF72* gene in patients diagnosed with sporadic ALS and FTD.

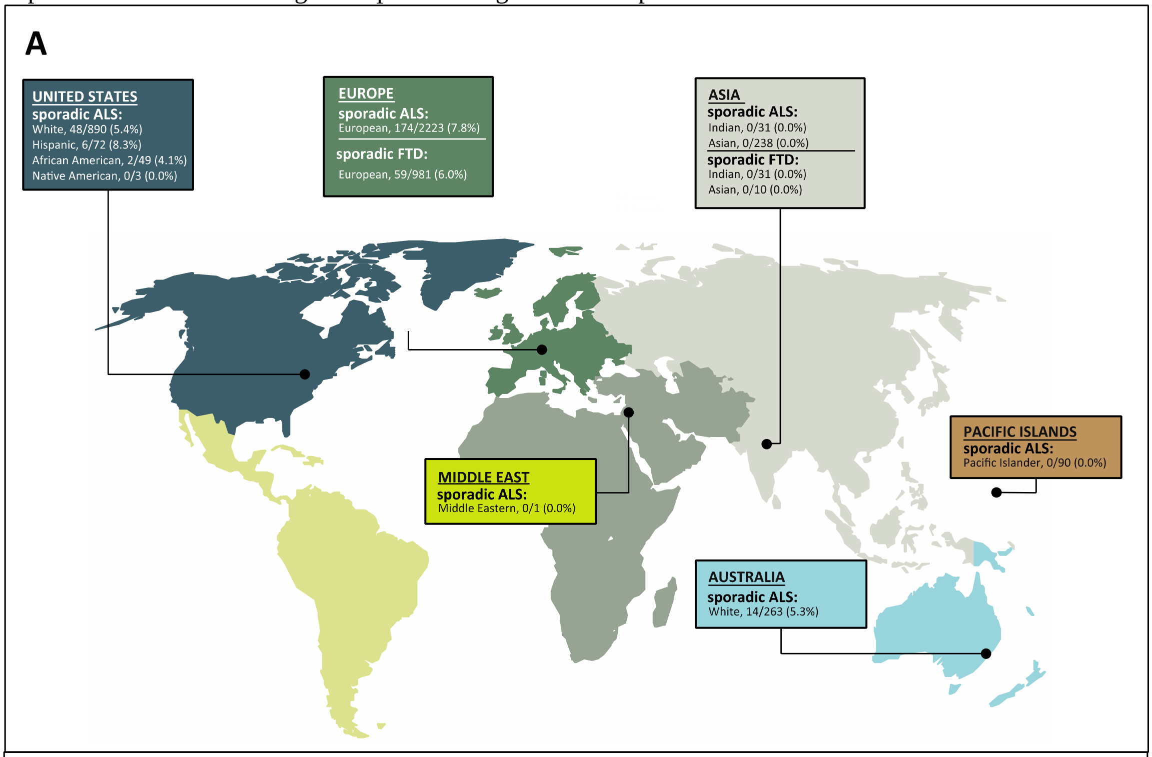

Supplementary Figure 2. Geographical distribution of the pathogenic GGGGCC hexanucleotide repeat expansion of the *C9ORF72* gene in patients diagnosed with familial ALS and FTD.

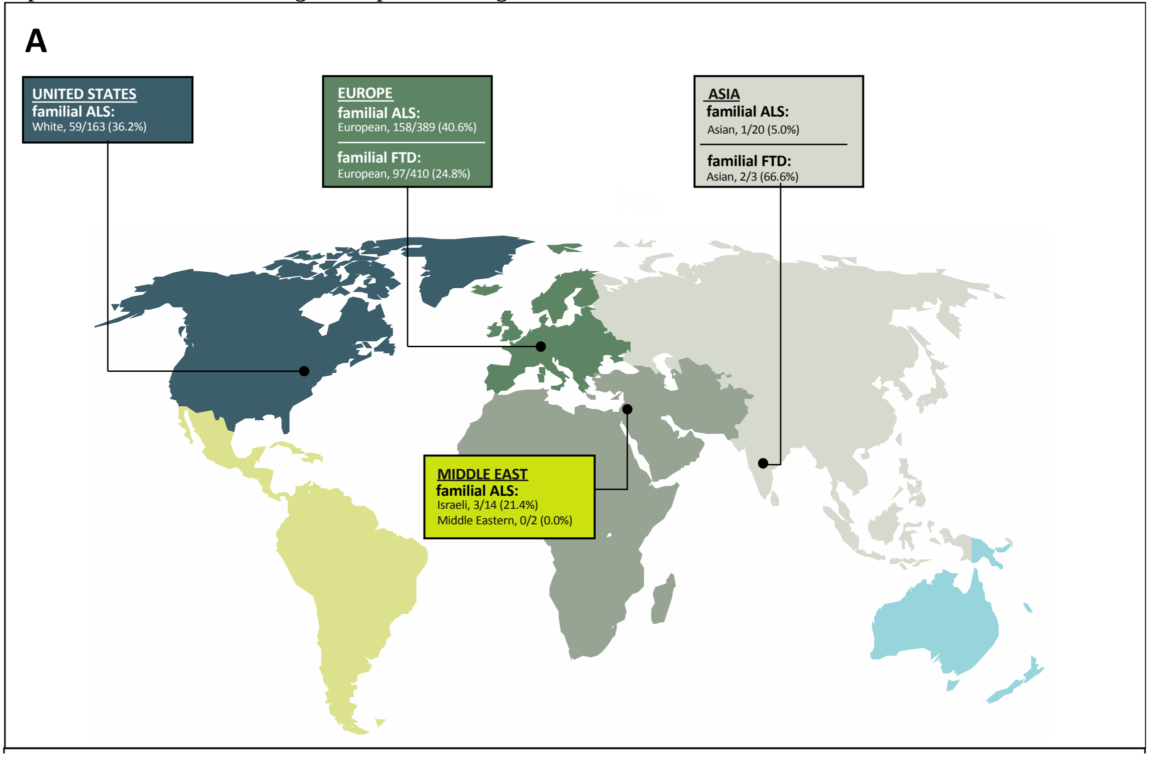

Supplementary Figure 3. Clade diagram showing age of the pathogenic GGCCCC hexanucleotide repeat expansion of the *C9ORF72* gene, and the approximate age of divergence of the Finnish, US and Italian populations carrying this mutation.

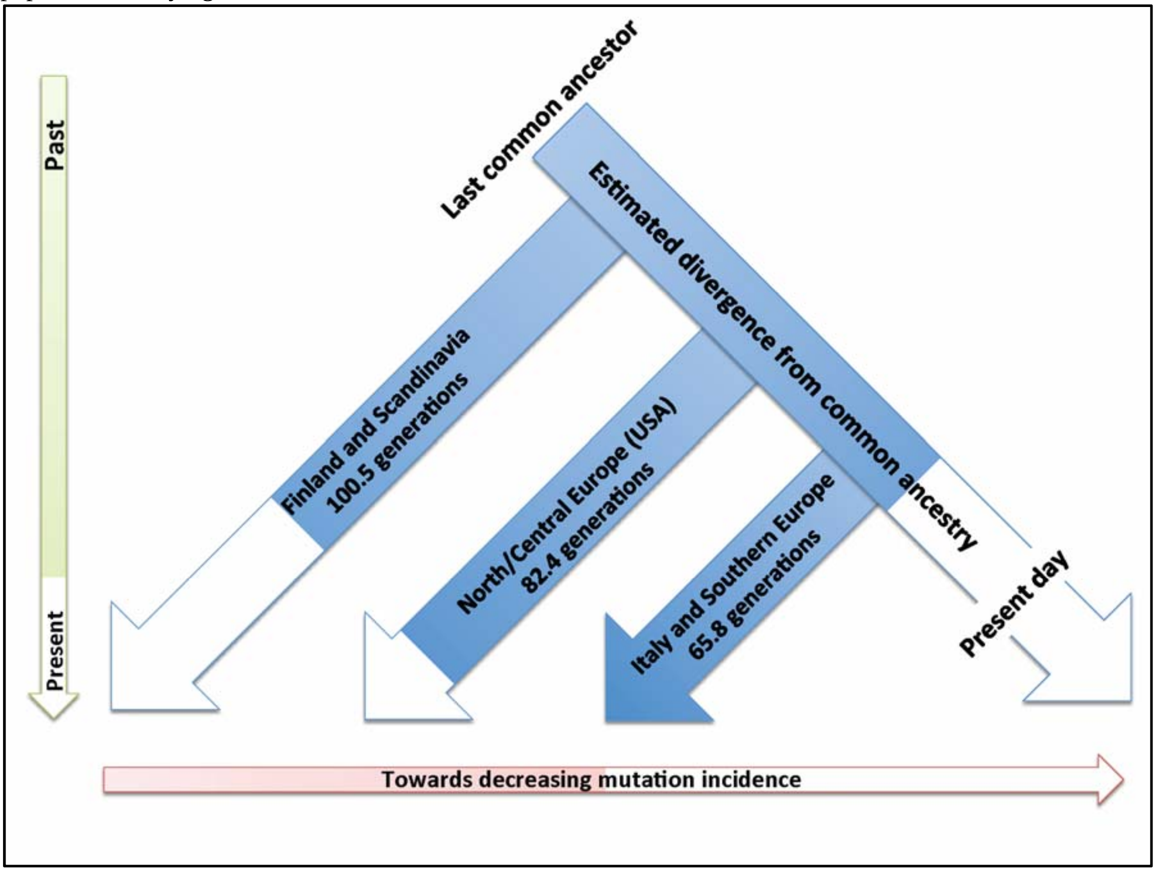

Supplementary Figure 4. Age-related penetrance of the GGGGCC hexanucleotide repeat expansion in the *C9ORF72* gene. (A) Age-related penetrance of patients presenting with ALS (n = 441) and FTD (n = 157). (B) Age related penetrance of male (n = 296) and female (n = 307) ALS and FTD patients. (C) Age-related penetrance of ALS patients presenting with bulbar-onset (n = 140) and limb-onset (n = 276) disease. (D) Age-related penetrance of FTD patients presenting with behavioural type (n = 73) and progressive non-fluent aphasia (PNFA, n = 9). Crosses represent censored-events (n = 5 neurologically normal individuals carrying the expansion). The dotted lines represent the age at which 50·0% of the cohort developed symptoms.

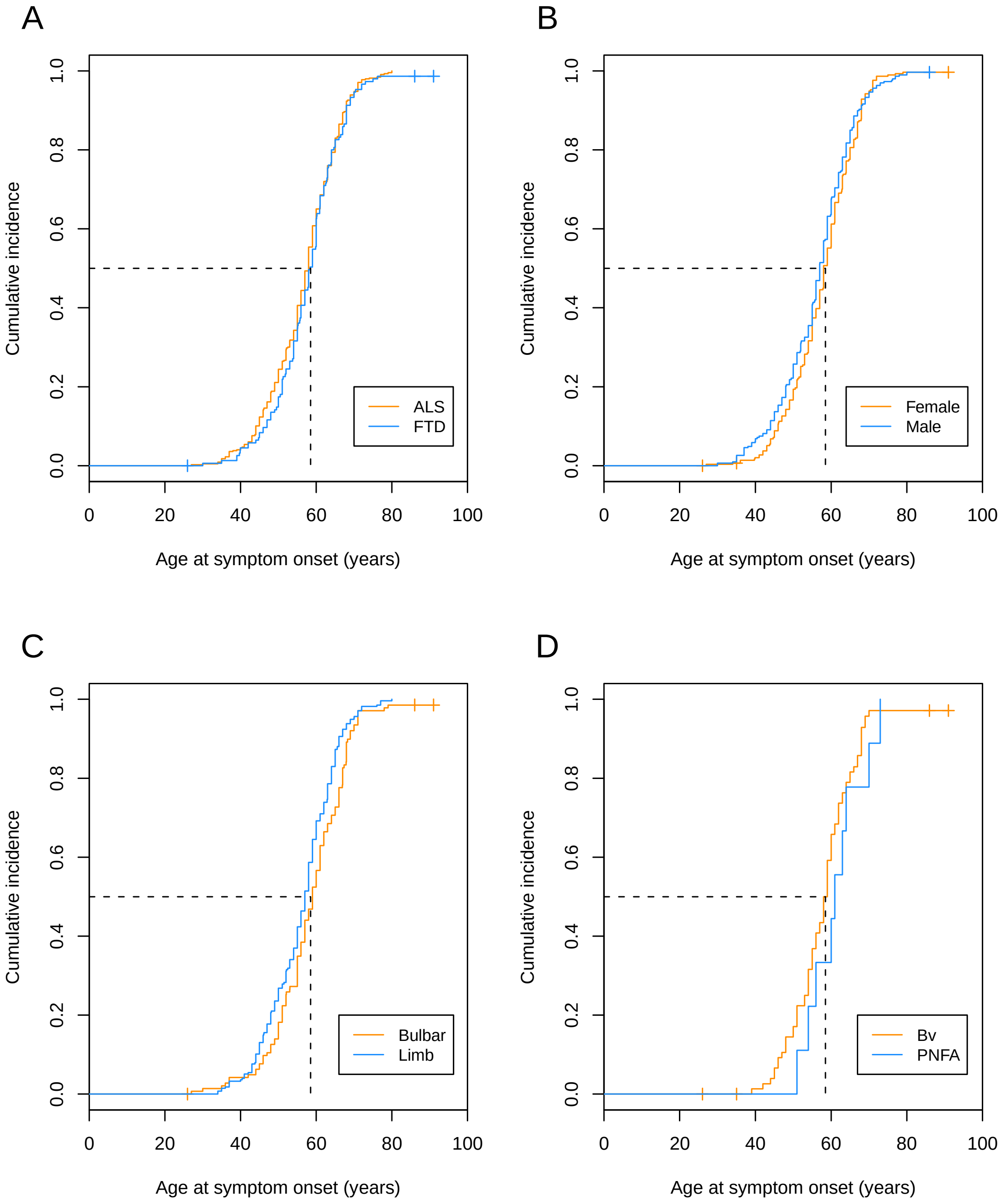

Supplementary references

1. Kobayashi H, Abe K, Matsuura T, et al. Expansion of Intronic GGCCTG Hexanucleotide Repeat in NOP56 Causes SCA36, a Type of Spinocerebellar Ataxia Accompanied by Motor Neuron Involvement. *Am J Hum Genet* 2011; **89**: 121-30.

2. Warner JP, Barron LH, Goudie D, et al. A general method for the detection of large CAG repeat expansions by fluorescent PCR. *J Med Genet* 1996; **33**: 1022-6.

3. Renton AE, Majounie E, Waite A, et al. A Hexanucleotide Repeat Expansion in *C9ORF72* Is the Cause of Chromosome 9p21-Linked ALS-FTD. *Neuron* 2011; **72**: 257-68.

4. Dejesus-Hernandez M, Mackenzie IR, Boeve BF, et al. Expanded GGGGCC Hexanucleotide Repeat in Noncoding Region of C9ORF72 Causes Chromosome 9p-Linked FTD and ALS. *Neuron* 2011; **72**: 245-56.

5. Byrne S, Elamin M, Bede P, et al. Cognitive and clinical characteristics of patients with amyotrophic lateral sclerosis carrying a C9orf72 repeat expansion: a population-based cohort study. *Lancet Neurol* 2012 DOI:10.1016/S1474-4422(12)70014-5.

6. Simón-Sánchez J, Dopper EG, Cohn-Hokke PE, et al. The clinical and pathological phenotype of C9orf72 hexanucleotide repeat expansions. *Brain* 2012. DOI: 10.1093/brain/awr353.

7. Snowden JS, Rollinson S, Thompson JC, et al. Distinct clinical and pathological characteristics of frontotemporal dementia associated with C9ORF72 mutations. *Brain* 2012. DOI: 10.1093/brain/awr355.

8. Stewart H, Rutherford NJ, Briemberg H, et al. Clinical and pathological features of amyotrophic lateral sclerosis caused by mutation in the C9ORF72 gene on chromosome 9p. *Acta Neuropathol* 2012. DOI: 10.1007/s00401-011-0937-5.

9. Gijselinck I, Van Langenhove T, van der Zee J, et al. A C9orf72 promoter repeat expansion in a Flanders-Belgian cohort with disorders of the frontotemporal lobar degeneration-amyotrophic lateral sclerosis spectrum: a gene identification study. *Lancet Neurol* 2012; **11**: 54-65.
